# Characterization of a Mouse Model of Emphysema Induced by Multiple Instillations of Low-Dose Elastase

**DOI:** 10.3389/fphys.2016.00457

**Published:** 2016-10-07

**Authors:** Milena V. Oliveira, Soraia C. Abreu, Gisele A. Padilha, Nazareth N. Rocha, Lígia A. Maia, Christina M. Takiya, Debora G. Xisto, Bela Suki, Pedro L. Silva, Patricia R. M. Rocco

**Affiliations:** ^1^Laboratory of Pulmonary Investigation, Carlos Chagas Filho Biophysics Institute, Federal University of Rio de JaneiroRio de Janeiro, Brazil; ^2^Department of Physiology and Pharmacology, Fluminense Federal UniversityNiteroi, Brazil; ^3^Laboratory of Cellular Pathology, Carlos Chagas Filho Biophysics Institute, Federal University of Rio de JaneiroRio de Janeiro, Brazil; ^4^Department of Biomedical Engineering, Boston UniversityBoston, MA, USA

**Keywords:** emphysema, elastance, collagen, elastic fiber, inflammation, cardiac function

## Abstract

Many experimental models have been proposed to study the pathophysiological features of emphysema, as well as to search for new therapeutic approaches for acute or chronically injured lung parenchyma. We aimed to characterize an emphysema model induced by multiple instillations of elastase by tracking changes in inflammation, remodeling, and cardiac function after each instillation. Forty-eight C57BL/6 mice were randomly assigned across two groups. Emphysema (ELA) animals received 1, 2, 3, or 4 intratracheal instillations of pancreatic porcine elastase (PPE, 0.2 IU) with a 1-week interval between them. Controls (C) received saline following the same protocol. Before and after implementation of the protocol, animals underwent echocardiographic analysis. After the first instillation of PPE, the percentage of mononuclear cells in the lung parenchyma increased compared to C (*p* = 0.0001). The second instillation resulted in hyperinflated alveoli, increased mean linear intercept, and reduced elastic fiber content in lung parenchyma compared to C (*p* = 0.0197). Following the third instillation, neutrophils and collagen fiber content in alveolar septa and airways increased, whereas static lung elastance was reduced compared to C (*p* = 0.0094). After the fourth instillation, the percentage of M1 macrophages in lungs; levels of interleukin-1β (IL-1β), keratinocyte-derived chemokine, hepatocyte growth factor (HGF), and vascular endothelial growth factor (VEGF); and collagen fiber content in the pulmonary vessel wall were increased compared to C (*p* = 0.0096). At this time point, pulmonary arterial hypertension was apparent, with increased diastolic right ventricular wall thickness. In conclusion, the initial phase of emphysema was characterized by lung inflammation with predominance of mononuclear cells, whereas at the late stage, impairment of pulmonary and cardiovascular functions was observed. This model enables analysis of therapies at different time points during controlled progression of emphysema. Accordingly, early interventions could focus on the inflammatory process, while late interventions should focus on restoring cardiorespiratory function.

## Introduction

Emphysema is a progressive disease characterized by airspace enlargement, followed by a decline in lung function (Vestbo et al., 1996). The pathogenesis of this disease is associated with an imbalance between elastase and anti-elastase activity (Janoff, 1985), rupture of alveolar walls (Suki et al., 2003), inflammation in the airways and lung parenchyma (Snider, 1985; Retamales et al., 2001), oxidative stress (MacNee, 2000), and apoptosis (Demedts et al., 2006). However, emphysema does not impair the lungs alone; it also produces extrapulmonary effects that contribute to its severity and mortality (Agustí et al., 2003; Fabbri et al., 2008). In this context, pulmonary arterial hypertension, which results from destruction of capillary vessel walls, may induce *cor pulmonale*, characterized by right ventricular hypertrophy and failure (Smith and Wrobel, 2014).

Several experimental models have been proposed to reproduce and explain the pathophysiology of emphysema, as well as to test new therapies (Wright et al., 2008; Fricker et al., 2014; Mercer et al., 2015; Vlahos and Bozinovski, 2015). Cigarette smoke (CS) is the most common cause of emphysema and has been used to evaluate cellular and molecular responses (Yao et al., 2010, 2012). However, the CS model does not reproduce the most severe, disabling aspects of the disease seen in humans, and requires several months of exposure (Churg and Wright, 2007), even though some groups have adopted acute CS exposure protocols to identify mediators and mechanisms involved in CS-induced inflammation (Mercer et al., 2015). Conversely, in the elastase-induced emphysema model, the disease develops rapidly after a single dose. Additionally, the severity of emphysema induced by elastase can be controlled according to enzyme dose (Snider et al., 1986; Snider, 1992). Nevertheless, repeated doses of elastase are required in order to develop systemic manifestations of emphysema. Indeed, Lüthje et al. showed, in NMRI mice, that multiple instillations of elastase caused weight loss, exercise intolerance, diaphragmatic dysfunction, and cardiovascular impairment (Lüthje et al., 2009). This model was adapted by Cruz et al., who reported, in C57BL/6 mice, that four instillations of elastase at 1-week intervals led to severe lung damage characterized by inflammation and remodeling, in addition to right ventricular dysfunction (Cruz et al., 2012). In both studies, analyses occurred after all elastase instillations, by which time the disease had progressed to the chronic stage. However, the pathophysiological consequences of each elastase administration during experimental emphysema development remain unknown. A detailed characterization of emphysema features after each elastase administration would lead to a better understanding of the multiple dose elastase-induced emphysema model.

Within this context, the aim of this study was to characterize a model of emphysema induced by multiple instillations of elastase through repeated analyses of pulmonary inflammatory and remodeling processes, as well as of cardiorespiratory function.

## Materials and methods

This study was approved by the Ethics Committee of the Health Sciences Centre, Federal University of Rio de Janeiro (CEUA-019). All animals received humane care in compliance with the “Principles of Laboratory Animal Care” formulated by the National Society for Medical Research and the U.S. National Research Council “Guide for the Care and Use of Laboratory Animals.” The present study followed the ARRIVE guidelines for reporting of animal research. Animals were housed in standard laboratory cages (12-h light/12-h dark cycles at a temperature of 23 ± 1°C), each one containing groups of three individuals. Mice had access to food and water *ad libitum*.

### Animal preparation and experimental protocol

**Figure 1:**
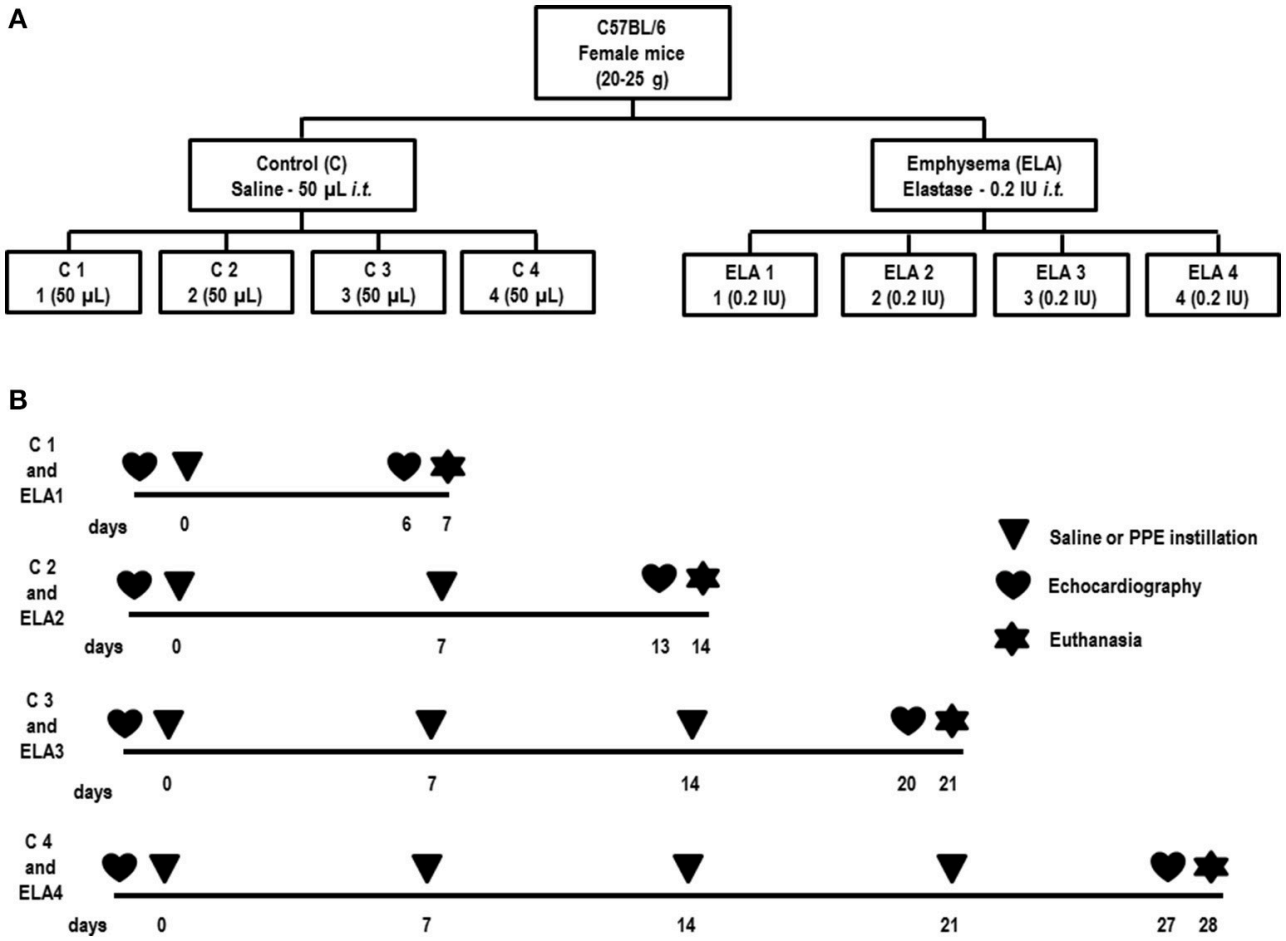
**Schematic flow chart (A) and timeline of study design (B)**. C group, control (animals that received 1, 2, 3, or 4 intratracheal instillations of saline at 1-week intervals). ELA1, single intratracheal instillation of pancreatic porcine elastase (PPE). ELA2, two instillations of PPE given 1 week apart. ELA3, three instillations of PPE at 1-week intervals. ELA4, four instillations of PPE at 1-week intervals.

Forty-eight female C57BL/6 mice (weight: 20–25 g, 2 months) were randomly assigned using closed sealed envelopes into two main groups: control (C) and emphysema (ELA). In the ELA group, animals were subdivided to receive a single intratracheal instillation of pancreatic porcine elastase [PPE (Sigma Chemical Co., St Louis, MO, USA) 0.2 IU in 50 μL saline—ELA1], two instillations with a 1-week interval (total dose, 0.4 IU PPE—ELA2), three instillations at 1-week intervals (total dose, 0.6 IU PPE—ELA3), and four instillations at 1-week intervals (total dose, 0.8 IU PPE—ELA4). C groups received saline (50 μL) using the same protocol (Figure 1). For all intratracheal instillations, which occurred in the laboratory between 9 and 12 a.m., mice were anesthetized with inhaled sevoflurane 2% (Sevorane®, Cristália, Itapira, SP, Brazil). A midline cervical incision (1 cm) was made to expose the trachea, and saline or PPE were instilled using a bent 27-gauge tuberculin needle. The cervical incision was closed with 5-0 silk suture, and the mice returned to their cages.

### Echocardiography

Twenty-four hours before the first instillation (INITIAL) and 24 h before euthanasia (FINAL), C and ELA animals underwent echocardiographic analysis. For echocardiographic assessment of cardiac function, the animals were anesthetized with inhaled sevoflurane 2%, shaved over the precordial region, and examined with a Vevo 770 system (VisualSonics, Toronto, ON, Canada) coupled to a 30-MHz transducer. Images were obtained from the parasternal, short-axis, and long-axis views. B-dimensional parasternal short axis views of both ventricles were acquired at the level of the papillary muscles to obtain left and right ventricular areas (Lang et al., 2006). M-mode images from the right outflow tract were obtained to evaluate right ventricular wall thickness. Pulsed-wave Doppler was used to measure pulmonary artery acceleration time (PAT), pulmonary artery ejection time (PET), and the PAT/PET ratio, an indirect index of pulmonary arterial hypertension (Thibault et al., 2010; Abbas et al., 2013). All parameters followed American Society of Echocardiography and European Association of Cardiovascular Imaging recommendations (Lang et al., 2015).

### Mechanical parameters

One week after the last instillation, the animals were premedicated with diazepam 10 mg/kg i.p. (Compaz®, Cristália, Itapira, SP, Brazil) anesthetized with thiopental sodium 20 mg/kg i.p. (Thiopentax®, Cristália, Itapira, SP, Brazil), tracheotomized, paralyzed with vecuronium bromide 0.005 mg/kg i.v. (Vecuron®, Cristália, Itapira, SP, Brazil), and ventilated with a constant flow ventilator (Samay VR15; Universidad de la Republica, Montevideo, Uruguay) using the following settings: rate 100 breaths/min, tidal volume (V_T_) 0.2 mL, and fraction of inspired oxygen (FiO_2_) 0.21. The anterior chest wall was surgically removed and a positive end-expiratory pressure of 2 cm H_2_O applied. Airflow and tracheal pressure (Ptr) were measured. Lung mechanics were analyzed by the end-inflation occlusion method. In an open chest preparation, Ptr reflects transpulmonary pressure (PL). Static lung elastance (Est,L) was determined by dividing lung elastic recoil pressure (Pel) by V_T_. Est,L was measured 10 times in each animal. All data were analyzed using ANADAT software (RHT-InfoData, Inc., Montreal, Quebec, Canada). All experiments lasted < 15 min.

### Lung histology

At the end of the experiment, laparotomy was performed and 1000IU of heparin (Hemofol®, Cristália, Itapira, SP, Brazil) was injected into the vena cava. The trachea was clamped at end-expiration, and the abdominal aorta and vena cava were sectioned, producing massive hemorrhage and terminal bleeding for euthanasia. The left lung was then removed, fixed in 3% buffered formalin, and embedded in paraffin; 4-μm thick slices were cut and stained with hematoxylin–eosin. Lung histology analysis was performed with an integrating eyepiece with a coherent system consisting of a grid with 100 points and 50 lines of known length coupled to a conventional light microscope (Olympus BX51, Olympus Latin America Inc., Brazil). The volume fraction of hyperinflated, collapsed, and normal pulmonary areas, the mean linear intercept (Lm), and the percentage of neutrophils in pulmonary tissue were determined by the point-counting technique across 10–20 random, non-overlapping microscopic fields at 200X, 400X, and 1000X magnification, respectively (Weibel, 1990; Hsia et al., 2010). In addition, inflammatory changes in the airway and pulmonary vessel wall were evaluated in a blinded fashion. The total number of cells was graded on a scale of 0–4, with 0 = absent; 1 = slight; 2 = mild; 3 = moderate; and 4 = severe.

The amount of collagen fibers (stained by the Picrosirius polarization method) was computed in alveolar septa, small airways, and pulmonary vessel walls. Elastic fiber content was computed in alveolar septa using Weigert's resorcin fuchsin method with oxidation. The images were generated by a microscope (Axioplan, Zeiss) connected to a digital camera (Sony Trinitron CCD, Sony, Tokyo, Japan) and fed into a computer through a frame grabber (Oculus TCX, Coreco, St. Laurent, QC, Canada) for offline processing. The thresholds for collagen and elastic fibers were established after enhancement of contrast up to the point where the fiber was easily identified as either birefringent (collagen) or black (elastic) bands at 400X magnification using Image Pro Plus 7.1 Software (Media Cybernetics, Silver Spring, MD, USA; Fullmer et al., 1974; Padilha et al., 2015; Henriques et al., 2016). The areas occupied by the elastic and collagen fibers were measured by digital densitometric recognition, divided by the tissue area of each zone of interest, and expressed as the percentage of elastic or collagen fiber in the alveolar septa, airways, or pulmonary vessel wall.

### Immunohistochemistry

Immunohistochemistry for macrophage subpopulations (M1 and M2 phenotypes) in lung tissue was done using iNOS rabbit anti-mouse polyclonal antibody (M1, catalog no. Rb-9242, Thermo Scientific) and arginase-1 rabbit anti-mouse polyclonal antibody (M2, catalog no. sc-20150, Santa Cruz Biotechnology). Antibodies were detected using a secondary antibody labeled with peroxidase (Histofine mouse MAX PO anti-rat and antirabbit, Nichirei Biosciences, Tokyo, Japan) followed by the chromogen substrate diaminobenzidine (Liquid DAB, Dakocytomation, USA, catalog no. K3468). Analysis was performed in 30 images of high-power fields (400X magnification) per slide, taken with an Evolution VR Cooled Color 13-bit digital camera (Media Cybernetics, Canada) and manually selected under a light microscope (Nikon Eclipse 400, Nikon Instruments Tokyo, Japan). The areas occupied by nucleated macrophages and cells with positive staining for the phenotype marker in each tissue were then calculated and expressed as fractional area occupied by positive cells. The images were analyzed using Image Pro Plus 7.1 (Media Cybernetics, Silver Spring, MD, USA).

### Enzyme-linked immunosorbent assay (ELISA)

Levels of keratinocyte-derived chemokine (KC, a mouse analog of interleukin-8), interleukin-1β (IL-1β), hepatocyte growth factor (HGF), and vascular endothelial growth factor (VEGF) in lung tissue were evaluated by ELISA using matched antibody pairs from PeproTech and R&D Systems (Minneapolis, MN, USA), according to manufacturer instructions. Results are expressed as pg/mL.

### Statistical analysis

The number of animals in each group was based on a previous study conducted in our laboratory (Cruz et al., 2012). A sample size of six animals per group would provide the appropriate power (1 − β = 0.8) to identify significant (α = 0.05) differences in mean linear intercept between C and ELA groups, taking into account an effect size d = 1.97, a two-sided test, and a sample size ratio = 1 (G ^*^- Power 3.1.9.2, University of Düsseldorf, Germany).

Normality and equality of variances were tested by the Kolmogorov–Smirnov test with Lilliefors correction and Levene's test, respectively. Parametric data were expressed as mean (SD) and non-parametric data as median (interquartile range). One-way ANOVA followed by Tukey's test was used to compare parametric data. A paired *t*-test was used to compare echocardiography parameters between INITIAL and FINAL at each time point. Statistical significance was established as *p* < 0.05. All tests were performed in the GraphPad Prism v6.07 statistical software package (GraphPad Software, La Jolla, California, USA).

## Results

Since no major differences were observed among the C groups at different time points, a single pooled control group was used to represent them.

### Lung mechanics

Static lung elastance was lower in ELA3 and ELA4 compared to the C (*p* = 0.0094 and 0.0038, respectively) and ELA1 (*p* = 0.0098 and 0.0028, respectively) groups (Table 1).

**Table 1:** **Lung mechanics, morphometry, and inflammatory cells**.

	**C**	**ELA1**	**ELA2**	**ELA3**	**ELA4**
Static lung elastance (cmH_2_O/mL)	35.9 ± 2.8	33.7 ± 2.9	29.5 ± 3.5	25.5 ± 2.8^*^^,^ ^**^	24.5 ± 2.4^*^^,^ ^**^
Normal (%)	99.7 ± 1.5	97.7 ± 1.0	81.2 ± 3.5^*^^,^ ^**^	64.7 ± 2.7^*^^,^ ^**^^#^	51.8 ± 3.8^*^^,^ ^**^^,^ ^#^^,^ ^†^
Collapse (%)	0.3 ± 1.5	2.3 ± 1.0	6.5 ± 1.7^*^^,^ ^**^	12.5 ± 0.7^*^^,^ ^**^^#^	17.2 ± 3.2^*^^,^ ^**^^,^ ^#^^,^ ^†^
Hyperinflation (%)	0.0 ± 0.0	0.0 ± 0.0	12.2 ± 2.6^*^^,^ ^**^	22.6 ± 2.1^*^^,^ ^**^^#^	30.9 ± 1.8^*^^,^ ^**^^,^ ^#^^,^ ^†^
Mononuclear cells (%)	31.0 ± 1.1	35.2 ± 0.5^*^	37.1 ± 1.2^*^	41.8 ± 1.5^*^^,^ ^**^^#^	46.2 ± 2.5^*^^,^ ^**^^,^ ^#^^,^ ^†^
Neutrophils (%)	1.4 ± 0.3	1.6 ± 0.3	2.0 ± 0.8	3.8 ± 1.7^*^	5.2 ± 1.5^*^^,^ ^**^^,^ ^#^
Inflammation score, airway	0 (0–1)	0.5 (0–1)	1 (0–2)	2.5 (2–3)^*^	3.5 (3–4)^*^^,^ ^**^^,^ ^#^
Inflammation score, pulmonary vessel wall	0.5 (0–1)	1 (0–1)	1 (1–2)	1.5 (1–2)	4 (2.75–4)^*^^,^ ^**^

*Fractional area of normal alveoli, collapsed alveoli, lung hyperinflation, mononuclear cells, neutrophils and airway and pulmonary vessel wall inflammation scores. C group, control (animals that received 1, 2, 3, or 4 intratracheal instillations of saline at 1-week intervals). ELA1, single intratracheal instillation of pancreatic porcine elastase (PPE). ELA2, two instillations of PPE given 1 week apart. ELA3, three instillations of PPE at 1-week intervals. ELA4, four instillations of PPE at 1-week intervals. Values are means ± SD of six animals in each ELA group and six animals in C group. For the inflammation score, values are median (interquartile range) of six animals in each ELA group and six animals in C group*.

* *Significantly different from C group (p < 0.05)*.

** *Significantly different from ELA1 group (p < 0.05)*.

# *Significantly different from ELA2 group (p < 0.05)*.

† *Significantly different from ELA3 group (p < 0.05)*.

### Lung morphometry

**Figure 2:**
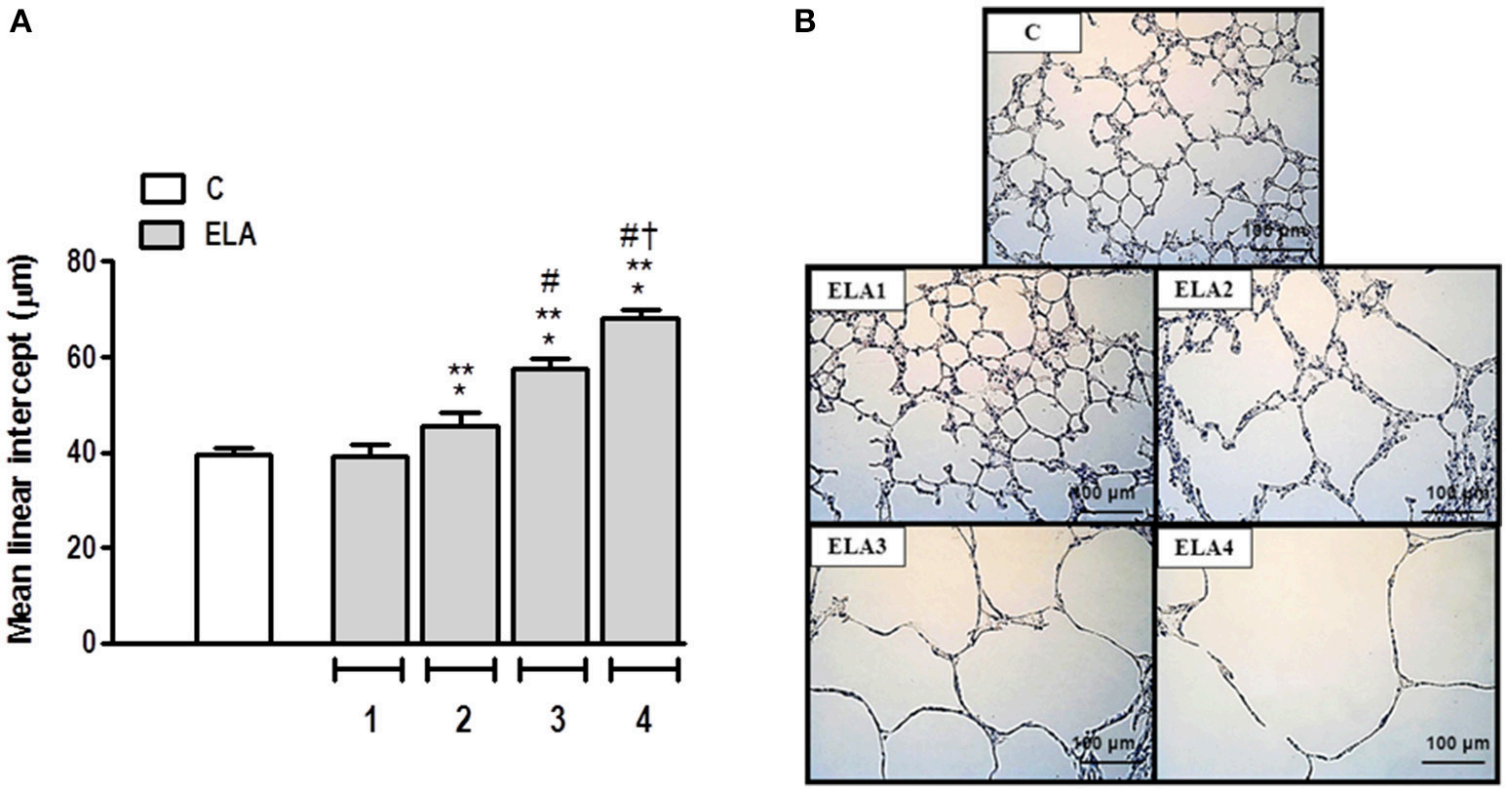
**Mean linear intercept (A) and representative photomicrographs of lung parenchyma stained with hematoxylin–eosin (H&E) (B)**. C group, control (animals that received 1, 2, 3, or 4 intratracheal instillations of saline at 1-week intervals). ELA1, single intratracheal instillation of pancreatic porcine elastase (PPE). ELA2, two instillations of PPE given 1 week apart. ELA3, three instillations of PPE at 1-week intervals. ELA4, four instillations of PPE at 1-week intervals. Values are means + *SD* of six animals in each group. ^*^Significantly different from C group (*p* < 0.05). ^**^Significantly different from ELA1 group (*p* < 0.05). ^#^Significantly different from ELA2 group (*p* < 0.05). ^†^Significantly different from ELA3 group (*p* < 0.05).

Mean linear intercept (Figure 2), fractional area of alveolar collapse, and hyperinflated alveoli (Table 1) increased in ELA2 compared to the C (*p* = 0.0039, 0.0017, 0.0002, respectively) and ELA1 groups (*p* < 0.0001, *p* = 0.0042, 0.0002, respectively).

### Elastic fiber content

**Figure 3:**
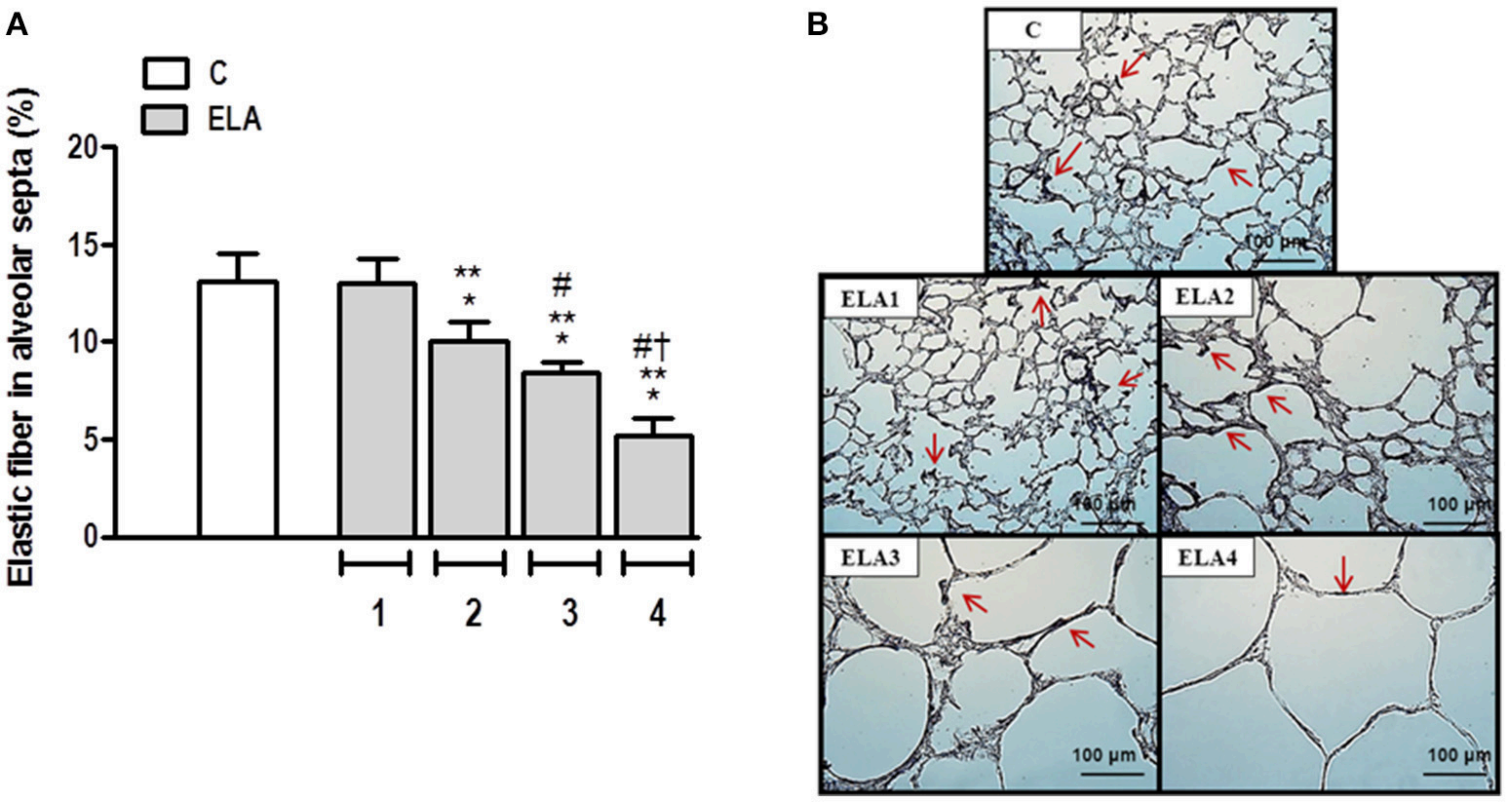
**Elastic fiber content in alveolar septa (A) and representative photomicrographs of lung parenchyma stained with Weigert's resorcin fuchsin method with oxidation (elastic fibers) (B)**. Arrows: Elastic fibers (stained black). C group, control (animals that received 1, 2, 3, or 4 intratracheal instillations of saline at 1-week intervals). ELA1, single intratracheal instillation of pancreatic porcine elastase (PPE). ELA2, two instillations of PPE given 1 week apart. ELA3, three instillations of PPE at 1-week intervals. ELA4, four instillations of PPE at 1-week intervals. Values are means + *SD* of six animals in each group. ^*^Significantly different from C group (*p* < 0.05). ^**^Significantly different from ELA1 group (*p* < 0.05). ^#^Significantly different from ELA2 group (*p* < 0.05). ^†^Significantly different from ELA3 group (*p* < 0.05).

The degree of elastolysis was evaluated by quantification of elastic fibers. Elastic fiber content decreased in ELA2 compared to the C and ELA1 groups (*p* = 0.0197 and 0.0132, respectively; Figure 3).

### Fibrosis in alveolar septa, airways, and pulmonary vessel wall

**Figure 4:**
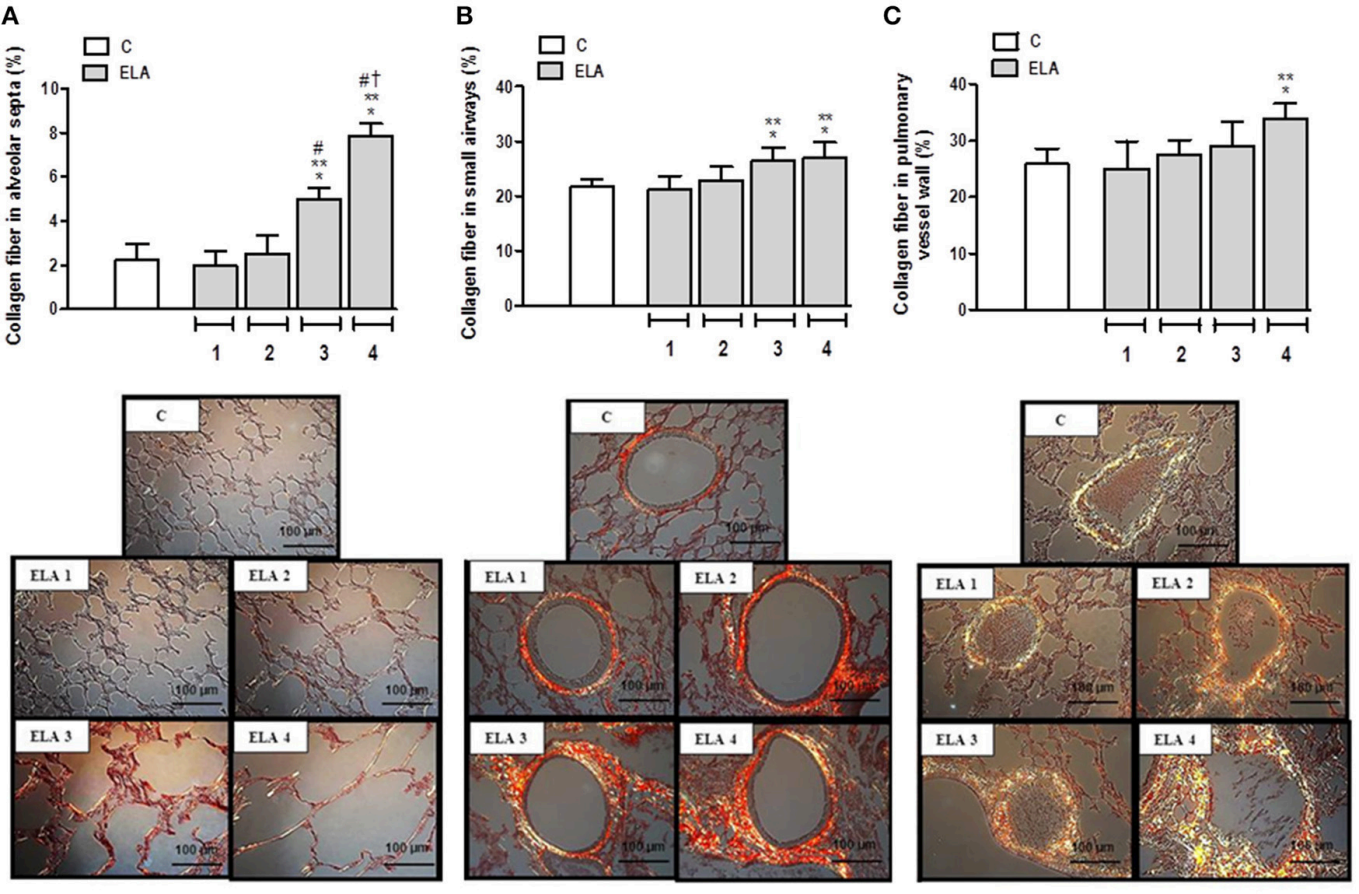
**Collagen fiber content and representative photomicrographs of alveolar septa (A), airways (B), and pulmonary vessel wall (C) stained with the Picrosirius-polarization method**. C group, control (animals that received 1, 2, 3, or 4 intratracheal instillations of saline at 1-week intervals). ELA1, single intratracheal instillation of pancreatic porcine elastase (PPE). ELA2, two instillations of PPE given 1 week apart. ELA3, three instillations of PPE at 1-week intervals. ELA4, four instillations of PPE at 1-week intervals. Values are means + *SD* of six animals in each group. ^*^Significantly different from C group (*p* < 0.05). ^**^Significantly different from ELA1 group (*p* < 0.05). ^#^Significantly different from ELA2 group (*p* < 0.05). ^†^Significantly different from ELA3 group (*p* < 0.05).

Collagen fiber content in alveolar septa was higher in ELA3 and ELA4 compared to the C (*p* = 0.0009 and *p* < 0.0001, respectively), ELA1 (*p* = 0.0002 and *p* < 0.0001, respectively), and ELA 2 (*p* = 0.0010 and *p* < 0.0002, respectively) groups. Additionally, the amount of collagen fiber in alveolar septa was higher in ELA4 than ELA3 (*p* = 0.0003; Figure 4A). In the airways, collagen fiber content was higher in ELA3 than in C (*p* = 0.0118) and ELA1 (*p* = 0.0015; Figure 4B). Collagen fiber content in the pulmonary vessel wall was higher in ELA4 than in the C and ELA1 groups (*p* = 0.0096 and 0.0034, respectively; Figure 4C).

### Correlations between lung morphometry, static lung elastance, and collagen and elastic fiber content

Interestingly, there was a correlation between increase in mean linear intercept and elastolysis (*p* < 0.0001), as well as with worsening of lung function (*p* < 0.0001; Figures S1A,B). There was also a strong correlation between increased deposition of collagen fibers and the increase in mean linear intercept (*p* < 0.001; Figure S1C).

### Lung inflammation

Lung inflammation was evaluated by measuring the fractional area of total cells as well as mononuclear cells and neutrophils in alveolar septa. Mononuclear cells increased in the lung parenchyma in ELA1 compared to C (*p* = 0.0001), while the percentage of neutrophils in lung tissue was higher in ELA3 than in controls (*p* = 0.0157; Table 1).

**Figure 5:**
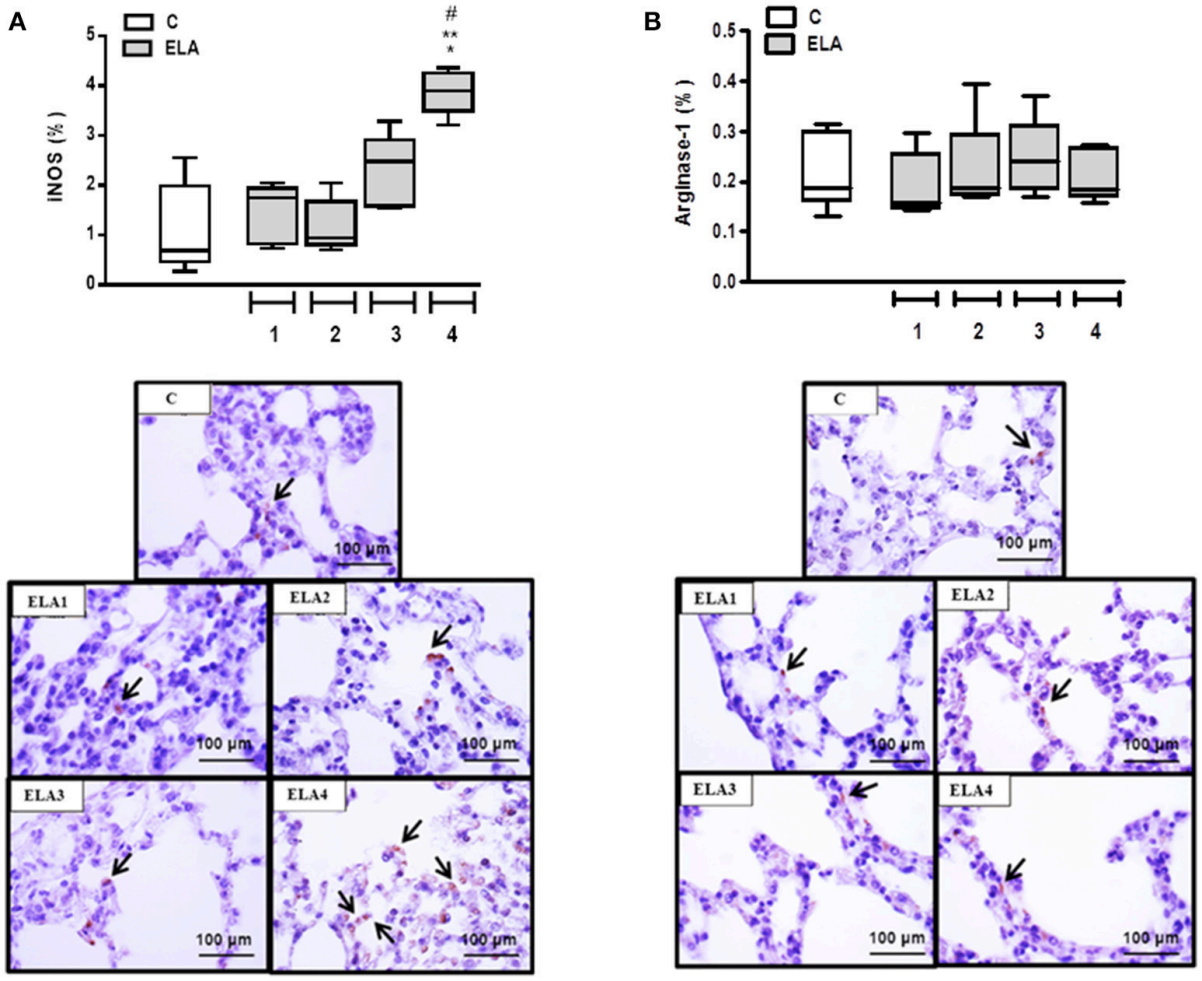
**Immunohistochemistry for iNOS (A) and arginase-1 (B)**. Note positive cells stained brown (arrows). C group, control (animals that received 1, 2, 3, or 4 intratracheal instillations of saline at 1-week intervals). ELA1, single intratracheal instillation of pancreatic porcine elastase (PPE). ELA2, two instillations of PPE given 1 week apart. ELA3, three instillations of PPE at 1-week intervals. ELA4, four instillations of PPE at 1-week intervals. Values are shown as box-plots (median, interquartile range, minimum, and maximum) of six animals in each group. ^*^Significantly different from C group (*p* < 0.05). ^**^Significantly different from ELA1 group (*p* < 0.05). ^#^Significantly different from ELA2 group (*p* < 0.05).

Alveolar macrophages play a crucial role in the pathogenesis of emphysema. They can be activated by a variety of extracellular signals to polarize into M1 macrophages (associated with antimicrobial response and inflammation) or M2 macrophages (associated with wound healing and resolution of inflammation). For a more in-depth analysis of lung inflammation, the M1 (iNOS-positive cells) and M2 (arginase-1-positive cells) subpopulations were quantified in this study. The M1 macrophage percentage was higher in ELA4 than C, ELA1, and ELA2 groups (*p* = 0.0014, 0.0036, 0.0005, respectively; Figure 5A). No significant difference was observed in the number of M2 macrophages between C and ELA groups, regardless of the time point of analysis (Figure 5B).

Additionally, the total number of inflammatory cells in the airway was increased in ELA3 compared to C and in ELA4 compared to the C, ELA1, and ELA2 groups. In the pulmonary vessel wall, the total number of inflammatory cells was higher in ELA4 compared to C and ELA1 animals (Table 1).

### Pro-inflammatory and pro-fibrogenic mediators in lung tissue

**Figure 6:**
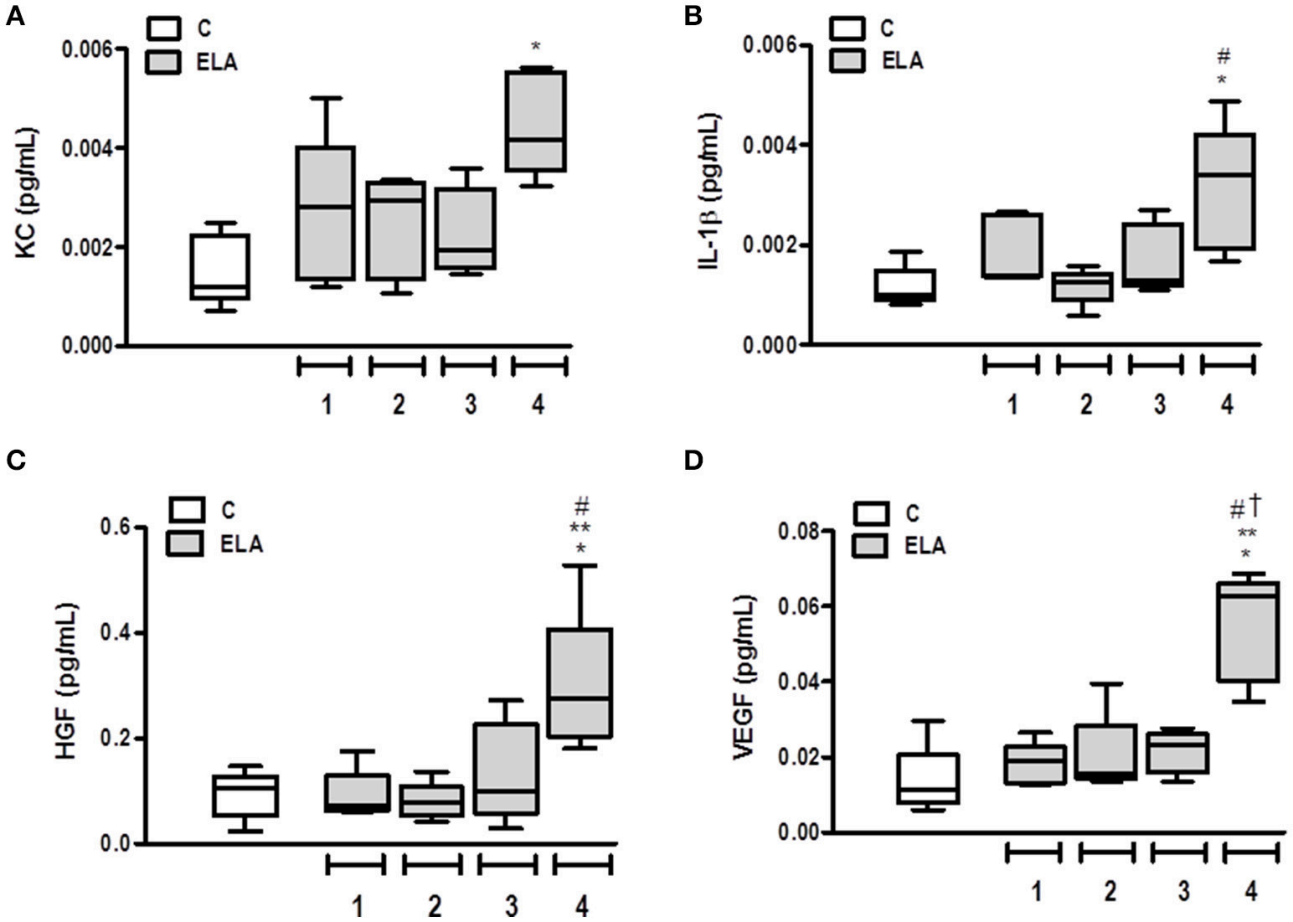
**Levels of keratinocyte-derived chemokine (KC, a mouse analog of interleukin-8) (A), interleukin (IL)-1β (B), hepatocyte growth factor (HGF) (C), and vascular endothelial growth factor (VEGF) (D) in lung tissue**. C group, control (animals that received 1, 2, 3, or 4 intratracheal instillations of saline at 1-week intervals). ELA1, single intratracheal instillation of pancreatic porcine elastase (PPE). ELA2, two instillations of PPE given 1 week apart. ELA3, three instillations of PPE at 1-week intervals. ELA4, four instillations of PPE at 1-week intervals. Values are shown as box-plots (median, interquartile range, minimum, and maximum) of six animals in each group. ^*^Significantly different from C group (*p* < 0.05). ^**^Significantly different from ELA1 group (*p* < 0.05). ^#^Significantly different from ELA2 group (*p* < 0.05). ^†^Significantly different from ELA3 group (*p* < 0.05).

Pro-inflammatory (KC and IL-1β and pro-fibrogenic (HGF and VEGF) mediators were measured in lung tissue. The KC level was higher in ELA4 than C (*p* = 0.0012), whereas the IL-1β level was increased in ELA4 compared to the C and ELA2 groups (*p* = 0.0094, 0.0148, respectively). HGF level was higher in ELA4 compared to the C, ELA1, and ELA2 groups (*p* = 0.0321, 0.0345, 0.0242, respectively), and VEGF level was increased in ELA4 compared to the C, ELA1, ELA2, and ELA3 groups (*p* = 0.0015, 0.0026, 0.0010, 0.0018, respectively; Figure 6).

### Cardiac function

**Figure 7:**
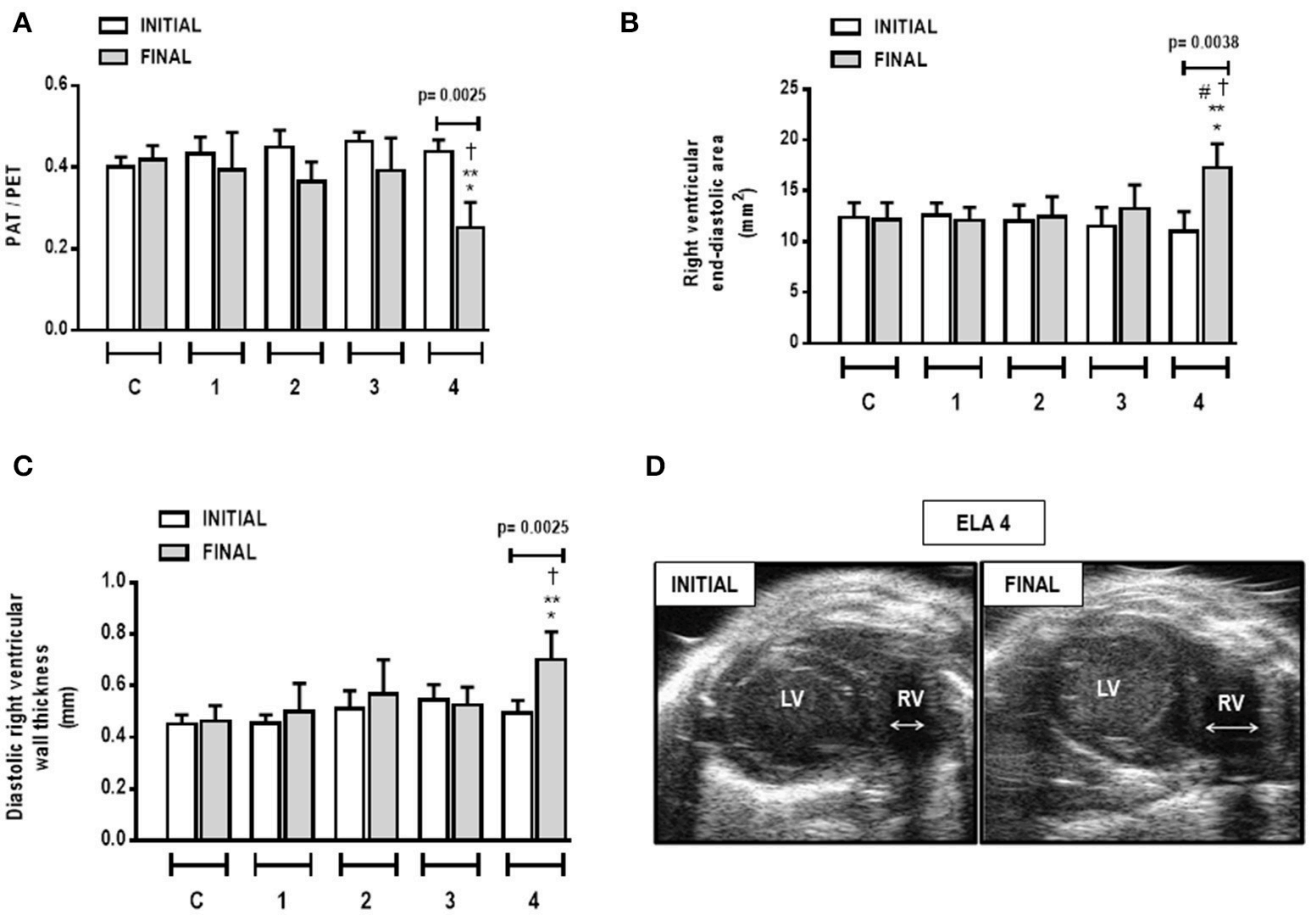
**Pulmonary artery acceleration time/pulmonary artery ejection time (PAT/PET) ratio (A), right ventricular end-diastolic area (B), diastolic right ventricular wall thickness (C), and short-axis B-dimensional views of both ventricles in ELA4 animals (D)**. LV, left ventricle; RV, right ventricle; INITIAL, before instillation of saline or PPE; FINAL, after instillation of saline or PPE. Values are means + *SD* of six animals in each group. ^*^Significantly different from C group (*p* < 0.05). ^**^Significantly different from ELA1-FINAL group (*p* < 0.05). ^#^Significantly different from ELA2-FINAL group (*p* < 0.05). ^†^Significantly different from ELA3-FINAL group (*p* < 0.05).

Cardiac function was evaluated using echocardiography. In ELA4, right ventricular end-diastolic area and diastolic right ventricular wall thickness were higher in FINAL compared to INITIAL (*p* = 0.0038 and 0.0025, respectively). Additionally, in FINAL, ELA4 animals exhibited increased right ventricular end-diastolic area compared to ELA1, ELA2, and ELA3 animals (*p* = 0.0115, 0.0325, 0.0106, respectively), as well as increased diastolic right ventricular wall thickness compared to the ELA1 and ELA3 groups (*p* = 0.0367, 0.0127, respectively). PAT/PET ratio was lower in FINAL than INITIAL in ELA4 (*p* = 0.0025). In FINAL, PAT/PET ratio was reduced further in ELA4 than in ELA1 and ELA3 (*p* = 0.0008 and 0.0381, respectively; Figure 7).

## Discussion

In the present study, we observed that: (1) the first instillation of PPE yielded an increased percentage of mononuclear cells in the lung parenchyma; (2) the second instillation resulted in hyperinflated alveoli, increased mean linear intercept, and reduced elastic fiber content in lung parenchyma; (3) the third instillation led to increased neutrophils and collagen fiber content in alveolar septa and airways, whereas static lung elastance was reduced; and (4) the fourth instillation yielded an increase in percentage of M1 macrophages in the lung, increased levels of IL-1β, KGF, HGF, and VEGF, and increased collagen fiber content in the pulmonary vessel wall. At this time point, pulmonary arterial hypertension, characterized by a reduced PAT/PET ratio, was present, with increased diastolic right ventricular wall thickness and right ventricular end-diastolic area. We have thus demonstrated that the current multiple elastase instillation model had a cumulative effect, starting with a mild inflammatory process followed by increased lung inflammation and changes in inflammatory cell profile, elastolysis, fibrosis in alveolar septa, airway, and pulmonary vessel wall, as well as cardiorespiratory functional impairment. Of particular note is the finding that, following the initial inflammation, airspace enlargement (a hallmark of emphysema) occurred simultaneously with loss of elastic fibers at week 2, whereas cardiopulmonary complications happened together with the appearance of collagen in the pulmonary vasculature.

Elastase has been widely used to induce emphysema in animals (Cruz et al., 2012; Craig et al., 2013; Kobayashi et al., 2013; Sandhaus and Turino, 2013; Antunes et al., 2014). However, lung morphological and functional changes have been usually evaluated at only one time point, which has hindered understanding of the full pathophysiology of this model. To our knowledge, this was the first study to evaluate the cumulative effect of elastase instillation on cardiorespiratory parameters.

After the first elastase instillation, a mild increase in mononuclear cells was observed, without lung damage or release of pro-inflammatory mediators and growth factors. Neutrophil infiltration and the increase in M1 macrophage percentage were present after the fourth elastase instillation, as were increased levels of KC and IL1-β. These changes may be associated with the presence of elastolysis, which was most intense at this time point (when the elastic fiber content reduced around 62%). In this context, elastin fragments act as chemoattractants for recruitment of macrophages and neutrophils (Houghton et al., 2006). Moreover, the elastolytic process occurs due to increased activity of neutrophils and macrophages, which release matrix metalloproteases, thus promoting elastic fiber rupture (Churg et al., 2012). After the second instillation of elastase, elastic fiber content was reduced even without neutrophil infiltration. We speculate that this loss of elastic fibers is associated with an increase in mononuclear cells releasing matrix metalloproteases (MMP), mainly MMP9, which has greater elastolytic capacity (Russell et al., 2002).

The mean linear intercept also increased with elastase instillations. This is consistent with previous studies that reported the development of structural changes after instillation of elastase (O'Donnell et al., 1999; Ito et al., 2004; Cheng et al., 2009; Hamakawa et al., 2011; Szabari et al., 2012).

Emphysema is characterized by changes in the organization and composition of extracellular matrix (Ito et al., 2005; Suki et al., 2012; Szabari et al., 2012; Takahashi et al., 2014; Robertoni et al., 2015). In an attempt to repair the damaged lung, new collagen fibers are resynthesized, but are relatively weak, and, because they can rupture, tend to reduce overall tissue stiffness and static lung elastance (Suki et al., 2003). Similarly, Ito et al. reported that increased total collagen fibers, including types I and III, led to an increase in compliance 21 days after elastase administration (Ito et al., 2005). A possible explanation for this apparent paradox is that the remodeling process assumes an abnormal character in emphysema, promoting the formation of weaker and disorganized collagen fibers (Hamakawa et al., 2011). In the present study, we observed a time lag between elastic fiber deterioration and collagen deposition. This could be explained by the fibroblasts, which are in a quiescent state during equilibrium. However, after injury, in an attempt to restore homeostasis, these cells become activated and convert into myofibroblasts (Stenmark et al., 2002), thus secreting collagen fibers (Wynn and Ramalingam, 2012).

Degradation of elastic fibers and remodeling of collagen allow rupture of septal walls, which, in this study, was most evident after the third and fourth instillations of elastase. This led to loss of elastic recoil and functional changes, which is consistent with other studies (Ito et al., 2005; Hantos et al., 2008; Hamakawa et al., 2011; Szabari et al., 2012).

HGF was increased only after the fourth instillation. In the lungs, HGF is produced by fibroblasts, and it stimulates epithelial cell proliferation after lung damage (Ohmichi et al., 1996; Sakamaki et al., 2002). Previous studies have shown that HGF levels increase 1 week after administration of a single dose of elastase (Shigemura et al., 2005). In addition, pulmonary endothelial cells are injured in emphysema, and can be the main source of VEGF. This growth factor is crucial for pulmonary vessel formation and development (Shalaby et al., 1995; Ferrara et al., 1996). Previous studies have shown that VEGF levels are reduced in emphysema models, suggesting endothelial cell injury (Kasahara et al., 2001; Cruz et al., 2012; Girón-Martínez et al., 2014). In contrast, in the present study, we observed an increase in VEGF in ELA4. We hypothesized that, with increased elastase administration, lung endothelial cells did indeed sustain more damage, but, since there are around 800 to 1000 capillaries for each alveolus (West, 2013), the remaining (undamaged) functional endothelial cells could increase VEGF levels. Additionally, we cannot rule out the possibility of crosstalk between HGF and VEGF (Cooper, 1992; Ferrara, 2004), whereby higher levels of HGF might increase VEGF synthesis.

Cardiovascular impairment was observed only after the fourth instillation, and was due to pulmonary damage. Previous studies have shown that the PAT/PET ratio is an important indirect indicator of pulmonary arterial hypertension (Beard et al., 1991; Jones et al., 2002; Thibault et al., 2010). In our study, the reduction in PAT/PET ratio can be attributed to increased collagen fiber content in lung vessels, which is in line with a previous study (Schreier et al., 2013). Together with vascular remodeling, arterial pulmonary hypertension can also be caused by hypoxic pulmonary vasoconstriction (Siebenmann and Lundby, 2015). This increased afterload induced morphological changes in the right ventricle, including in its area and wall thickness. Such changes characterize *cor pulmonale*, one of the main conditions that lead to death in patients with emphysema (Kawut et al., 2014).

This study has some limitations. First, no animal model is expected to reproduce all features of emphysema as it occurs in humans, since the natural history of the condition is not observed in non-human animals, and the various comorbidities that are often observed in humans are also absent in animals. Each choice of animal model has its own benefits and deficiencies. Nevertheless, there is a great need to develop animal models closely resembling various aspects of human emphysema to test different drugs and therapies that might reduce inflammation and improve lung repair. Therefore, a better understanding of the morphological and functional changes that occur during the course of our model of emphysema will enable us to test therapies early and late in the course of this disease. Second, each intratracheal administration contained 0.2 IU of PPE, which could be considered a low dose compared to those used in previous studies. To clarify whether a single 0.8-IU dose of PPE would result in similar effects, additional experiments were performed in 10 animals, but all died 3 days after instillation. Third, analysis of the degradation and remodeling of extracellular matrix components was focused on collagen and elastic fiber content, and not on organization of other components. Fourth, only female mice were included in the sample. This was a deliberate choice based on the previous work of Cruz et al. (2012), in which female mice were used to localize the site of bone marrow injections whose cells were derived from male mice. Further studies using both female and male animals are needed to elucidate whether sex-specific responses to the elastase model exist. Finally, the current model was not compared with other models of CS-induced or elastase-induced emphysema, since our aim was to evaluate the temporal evolution of pulmonary inflammatory, airway remodeling, and cardiorespiratory function in a model of emphysema induced by multiple instillations of low-dose elastase.

In conclusion, the initial phase of elastase-induced emphysema in mice was characterized by lung inflammation with predominance of mononuclear cells, whereas at the late stage, impairment of pulmonary and cardiovascular functions was observed. This model enables analysis of therapies at different time points during the clinical course of emphysema. Given our findings, we suggest that early interventions for emphysema management could focus on the inflammatory process, while late interventions should focus on restoring cardiorespiratory function.

## Author contributions

MO, GP, NR, DX, PS, and PR conceived and designed the experiments. MO, GP, NR, LM, CT, DX, and SA performed the experiments and analyzed the data. MO, SA, BS, PS, and PR coordinated data collection and data quality assurance. MO, SA, BS, PS, and PR participated in the first draft of the manuscript. All authors participated in the writing process of the manuscript and read and approved the final version.

## Funding

This study was supported by Conselho Nacional de Desenvolvimento Científico e Tecnológico/Ministério da Saúde/DECIT (469716/2014-2, 465064/2014-0, and 400462/2014-1 to PR) and Fundação Carlos Chagas Filho de Amparo à Pesquisa do Estado do Rio de Janeiro (E-26/103.118/2 to PR).

### Conflict of interest statement

The authors declare that the research was conducted in the absence of any commercial or financial relationships that could be construed as a potential conflict of interest.
