# Supplementary material for: Characterization of a Mouse Model of Emphysema Induced by Multiple Instillations of Low-Dose Elastase
Source: Front Physiol. 2016 Oct 7;7:457. doi: 10.3389/fphys.2016.00457 (PMC5054025; doi:10.3389/fphys.2016.00457)
Supplement: Supplementary file 1 [file DataSheet1.docx]

**Characterization of a Mouse Model of Emphysema Induced by Multiple Instillations of Low-Dose Elastase**

Milena V. Oliveira¹^#^, Soraia C. Abreu¹^#^, Gisele A. Padilha¹, Nazareth N. Rocha¹^,3^, Lígia A. Maia¹, Christina M. Takyia², Debora G. Xisto¹, Bela Suki^4^, Pedro L. Silva¹, Patricia R. M. Rocco¹^#^

^1^Laboratory of Pulmonary Investigation, ²Laboratory of Cellular Pathology, Carlos Chagas Filho Biophysics Institute, Federal University of Rio de Janeiro, RJ, Brazil, ^3^Department of Physiology and Pharmacology, Fluminense Federal University, Niteroi, RJ, Brazil, ^4^Department of Biomedical Engineering, Boston University, Boston, MA, USA.

^#^These authors contributed equally to this work

*Corresponding author:

Prof. Patricia Rieken Macedo Rocco, M.D., Ph.D.

Laboratory of Pulmonary Investigation, Carlos Chagas Filho Biophysics Institute, Federal University of Rio de Janeiro, Centro de Ciências da Saúde, Avenida Carlos Chagas Filho, s/n, Bloco G-014, Ilha do Fundão, Rio de Janeiro, RJ 21941-902 – Brazil.

Phone: (+5521) 3938-6530 Fax: (+5521) 2280-8193

E-mail: [prmrocco@biof.ufrj.br](mailto:prmrocco@biof.ufrj.br)

**SUPPLEMENTARY INFORMATION**

Figure S1: Spearman correlation between mean linear intercept and elastic fiber in alveolar septa (A), static lung elastance (B), and collagen fiber in alveolar septa (C). Circle represents ELA1 group, square represents ELA 2 group, up-pointing triangle represents ELA3 group, and down-pointing triangle represents ELA4 group. The *r* value represents the correlation coefficient, and *p*, the respective p-value. Statistical significance was accepted at p < 0.05.

**A**

**B**

**C**
